# Eléments traces dans le sérum des enfants malnutris et bien nourris vivants à Lubumbashi et Kawama dans un contexte d'un environnement de pollution minière

**DOI:** 10.11604/pamj.2016.24.11.9236

**Published:** 2016-05-04

**Authors:** Aimée Mudekereza Musimwa, Gray Wakamb Kanteng, Hermann Tamubango Kitoko, Oscar Numbi Luboya

**Affiliations:** 1 Département de Pédiatrie, Faculté de Médecine Université de Lubumbashi, République Démocratique du Congo

**Keywords:** Eléments trace, malnutrition, enfant, Lubumbashi, Malnutrition, trace elements, trace metals elements, child, Lubumbashi

## Abstract

**Introduction:**

La place des éléments traces métalliques essentiels en nutrition humaine ne peut plus être ignorée. Les déficits d'apports, les carences secondaires souvent sous – estimées, et les carences iatrogènes font le lit de pathologies telles que les infections et autres. D'où leurs dosages ont une importance particulière pour en évaluer la gravité et faciliter une prise en charge précoce ou améliorer le régime alimentaire. Cette étude a eu pour objectif de déterminer le profil sanguin en éléments traces (cuivre, sélénium, zinc, fer, chrome, cobalt, etc) chez les enfants malnutris et biens nourris dans un milieu minier à Lubumbashi.

**Méthodes:**

Trois cents onze cas ont été colligés, 182 malnutris et 129 biens nourris, dans une étude descriptive transversale, effectuée de juillet 2013 à décembre 2014. Pour lequel un échantillonnage exhaustif a été réalisé. Le dosage des métaux dans le sérum s'est fait à l’ ICP-OES (spectrométrie de masse à plasma gon induit) au laboratoire de l'Office Congolais de Contrôle de Lubumbashi.

**Résultats:**

Les oligoéléments essentiels (cuivre, zinc, sélénium et fer) se retrouvent à des concentrations très basses chez les enfants malnutris comme chez les biens nourris. L'arsenic, le cadmium, le magnésium et le manganèse se présentent à des concentrations normales par rapport aux valeurs de références chez les enfants biens nourris. L'antimoine, le chrome, le plomb et le cobalt se retrouvent élevés chez les malnutris et biens nourris. Le nickel est normal chez les malnutris et les biens nourris. Le magnésium, manganèse se sont présentés à des taux très bas chez les enfants malnutris.

**Conclusion:**

Les enfants malnutris et biens nourris présentent une malnutrition aux oligo-éléments essentiels associés aux éléments traces métalliques. Ce qui permet de supposer qu'une carence en micronutriments essentiel favorise l'absorption des métaux lourds.

## Abstract (en)

**Introduction:**

The role of trace metals elements in human nutrition can no longer be ignored. Deficiency caused by inadequate dietary intake, secondary deficiencies often under - estimated, and iatrogenic deficiencies lead to pathologies such as infections and others. For this reason their dosages are particularly important to assess disease severity and to facilitate early treatment or improve patient's diet. The aim of this study was to determine trace elements profile in blood (copper, selenium, zinc, iron, chromium, cobalt, etc.) among malnourished and well-nourished children in a mining community in Lubumbashi.

**Methods:**

Three hundred eleven cases have been collected, 182 malnourished children and 129 well-nourished children in a cross-sectional descriptive study conducted from July 2013 to December 2014. Exhaustive sampling was performed. Metal determination in serum was performed using Inductively Coupled Plasma Spectroscopy (ICP-OES/MS) in the laboratory at Congolese Control Office in Lubumbashi.

**Results:**

Essential trace elements (copper, zinc, selenium and iron) were found at very low concentrations in both the malnourished and well-nourished children. Arsenic, cadmium, magnesium and manganese concentrations were normal compared with reference values in well-nourished children Antimony, chromium, lead and cobalt levels were high in both the malnourished and well-nourished children. Nickel level was normal malnourished and well-nourished children. Magnesium, manganese were found in very low levels in malnourished children.

**Conclusion:**

Both the malnourished and well-nourished children suffer from deficiencies of essential trace elements associated with trace metals elements This allows to assume that essential micronutrients deficiency promotes the absorption of heavy metals.

## Introduction

L'exposition aux métaux toxiques et la carence en oligo-éléments constituent un véritable problème de santé publique en raison de la qualité nutritionnelle insuffisante. Éléments naturellement présents dans les sols dont certains sont indispensables aux plantes. Ils font partie des oligo-éléments et des Éléments Traces. On utilise également l'expression métaux lourds, qui correspond à une définition physique (masse volumique supérieure à 5 g/cm3) ou bien oligo-éléments. Les ETM les plus connus pour leur dangerosité sont le plomb (Pb), le mercure (Hg), le cadmium (Cd), le chrome (Cr), le cuivre (Cu), le nickel (Ni), le zinc (Zn). Il faut ajouter à cette liste l'arsenic (As) et le sélénium (Se), qui ne sont que des éléments Traces et pas des métaux [1]. Les oligo-éléments, appelés aliments protectifs, sont des éléments minéraux nécessaires au bon fonctionnement de l'organisme, en très faible quantité? 1mg/kg de poids corporel; d'où le nom « d’éléments traces » [2].

La place des oligo-éléments essentiels en nutrition humaine ne peut plus être ignorée. Les déficits d'apports, les carences secondaires souvent sous - estimées, et les carences iatrogènes font le lit de pathologies telles que les infections et autres [3]. Les carences en micronutriments présentent des interactions complexes qui conduisent au cercle vicieux de la malnutrition et des infections [4]. En R.D Congo, les taux de malnutrition infantile restent très élevés dans les provinces qui dépendent de l′industrie minière comparativement aux niveaux observés dans les provinces de l′Est en proie aux conflits [5]. Dans ces régions minières, une exposition soutenue aux facteurs nuisibles environnementaux ou aux déchets miniers peuvent endommage le développement physique et mental de l'enfant. Le cerveau d′un enfant est plus vulnérable aux dommages causés par des agents toxiques [6]. Les effets des produits chimiques environnementaux sur la santé des enfants ont été signalés largement, avec la majorité d'auteurs se concentrant sur les effets nocifs sur le système nerveux central [7]. En outre, le comportement des enfants de porter la main à la bouche ainsi que de jouer près du sol augmente également leur probabilité d′exposition [8, 9]. Plusieurs études ont démontré récemment, les effets indésirables liés à l′exposition aux éléments traces métalliques, sur la santé des enfants, où les principales conséquences ont été les déficits de l'enseignement, de l′attention et une atteinte rénale [10–12]. La province du Katanga région minière, se retrouve en deuxième position après la province du Maniema où la malnutrition et la mortalité infantile sont les plus élevées en RD Congo [13]. A Lubumbashi aucune étude n'a été réalisée chez l'enfant sur le taux plasmatique des oligo-éléments et à fortiori chez l'enfant malnutri à Lubumbashi. Banza et al 2012[14] ont révélé des concentrations urinaires des éléments traces métalliques anormalement élevées chez des personnes vivant dans le voisinage des industries minières et métallurgiques du sud-est du Katanga. A l'exception du nickel, les concentrations urinaires de ces éléments traces métalliques étaient significativement plus élevées chez les habitants des environs des activités minières ou industrielles (Sud-est du Katanga) que chez ceux qui vivent dans la région n'ayant pas ce genre d'activités ou à Kamina.

La réalisation de cette étude se justifie avant tout par le besoin de connaitre les taux des éléments dont le dosage est offert dans le cadre du dépistage par ICP-OES (spectrométrie de masse à plasma à plasma d'argon induit). Le manque de données locales concernant les valeurs de référence d’éléments moins fréquemment dosés tels que le cobalt, le manganèse, le magnésium, le chrome, le cadmium, le cuivre, le zinc, le plomb, le nickel et autres; d'où l'intérêt de l’étude.

Les taux plasmatiques en protéines et en éléments minéraux sont logiquement influencés par les apports alimentaires au niveau des ménages et par l'exposition aux polluants et toxiques divers engendrés par la production, le stockage et le transport incontrôlés. A Lubumbashi, les études fiables portant sur les minéraux dans le plasma sont rares. D'où la question de savoir: quel serait le profil plasmatique biochimique en oligo-éléments chez l'enfant de 0 à 5 ans vivant à Lubumbashi et ses environs.

L'objectif principal de cette étude est de déterminer sur le profil sanguin en minéraux chez les enfants mal nourris et bien nourris à Lubumbashi.

## Méthodes

Il s'agit d'une étude descriptive transversale couvrant la période du 01 juillet 2013 au 31 décembre 2014, effectuée dans la zone urbaine et péri urbaine de Lubumbashi, au sud-est de la République Démocratique du Congo. Il s'agit de l'hôpital Général de Référence Jason Sendwe, l'hôpital Général de Référence Kisanga, l'hôpital Militaire Camp Vangu, l'hôpital de Référence Mamba 2 et dans le village Kawama situé à 30 km de la ville de Lubumbashi. Notre étude a porté sur un échantillonnage exhaustif de 311enfants âgés de 06 à 59 mois, soit 182 malnutris nouvellement admis au centre de réhabilitation ou de prise en charge pour MAS(malnutrition aigüe sévère) et n'ayant pas encore reçu de traitement de prise en charge de la malnutrition, et 129 en bonne état nutritionnel recrutés dans des dispensaires de pédiatrie des hôpitaux ci haut cités.

Le diagnostic de la malnutrition a été défini selon les critères de l'Organisation Mondiale de la Santé (OMS 2006) par: l'indice poids-âge inférieur à?2 écarts types et périmètre brachial inférieur à 115mm ou œdèmes bilatéraux et signes cliniques de malnutrition. Un prélèvement a été effectué chez chacun d'entre eux. Le sang (5ml) recueilli sera transporté au laboratoire de l'OCC (Office Congolais de Contrôle) pour le traitement et la lecture par la spectrométrie d'absorption atomique et à l'ICP (ICP-OES) utilisant le plasma par couplage inductif. Un spectromètre à Emission optical OES de la marque Perkin Elmer de la série 8300 à double vision a été employé.

Les données ont été saisies et analysées sur ordinateur à l'aide du logiciel Epi info version 3.5.2. L'analyse statistique a porté sur des comparaisons univariées. Chaque paramètre était calculé à un intervalle de confiance (IC) à 95%.

Le protocole du travail a été soumis et approuvé au Département de Pédiatrie des Cliniques Universitaires de Lubumbashi. Nos prélèvements ont été effectués sous le consentement verbal libre et éclairé des parents de chaque enfant, après une explication brève du but de notre étude.

## Résultats

Les données du Tableau 1 montre que l'antimoine, le nickel, le plomb, le chrome et le cobalt sont à des concentrations très élevés chez l'enfant bien nourris. Le fer, le cuivre, le zinc et sélénium se retrouvent à des concentrations sériques tr ès basses sinon normales. A l'analyse du Tableau 2 montre que le cobalt, le chrome, l'antimoine et le plomb sont à des concentrations élevées. Le fer, le cuivre, le zinc et le sélénium sont à des concentrations médianes très basses.

**Tableau 1:** Valeur des métaux chez l'enfant biens nourris

Eléments	VNµg/L	Médiane	Mode	Appréciation
**As**	< 15	0,0001	0,0001	N
**cd**	<2	0,358	0,0001	N
**Co**	<0,5	1,49	0,0001	+ +
**Cr**	<0,3	6,404	6,02	+ + +
**Cu**	700-1550	68,38	102,5	–
**Fe**	600-1300	274	367,1	–
**Mg**	18-22	18,12	10,00	N
**Mn**	0,5 -2,5	2,619	0,85	N
**Ni**	< 5	5,65	6	+
**Pb**	< 5	10,9	0,0001	N
**Sb**	< 0,20	5,7	0,0001	+ + +
**Se**	60-120	7,04	0,0001	–
**Zn**	600 - 1300	18,81	18,81	–

VN: valeur normale; N: normal; +:augmenté; + + +: très augmenté; -:bas

**Tableau 2:** Valeur des métaux chez les enfants mal nourris

Eléments	VN	Médiane	Mode	Appréciation
As	<15 µg/l	1,895	3,123	N
cd	<2µg/l	0,1025	0,2	N
Co	<0,5µg/l	2,478	1,328	+
Cr	<0,3µg/l	2,726	1,369	+
Cu	700-1550µg/l	91,38	73,39	–
Fe	600-1300	159,5	78,53	–
Mg	18-22	0,8715	0,018	–
Mn	0,5 -2,5	0,0002	0,0001	N
Ni	<5	1,7315	0,0001	N
Pb	< 5	10,69	6427	+
Sb	< 0,20	1,1545	0,37	+
Se	60 -120	0,0001	0,0001	–
Zn	600 - 1300	23,76	15,58	–

VN: valeur normale; N: normal; +:augmenté; + + +: très augmenté; -:bas

## Discussion

### Arsenic

Dans cette étude, nous avons trouvé des médianes très faibles dans nos deux populations (mal nourris et biens nourris) soit 1,895 et 0,0001 µg/l. Zheng et al, 2012 [15] trouvent que l′exposition à l′arsenic était associée à un risque accru de maladie pulmonaire. Les troubles des comportementaux et neuropsychologiques ont également été liés à l′exposition à des concentrations élevées en nickel chez l'enfant [16, 17].

Contrairement à l’étude menée par l'Institut National de Santé Publique du Québec 2001 [18], cette étude a enregistré des valeurs faibles. Dans l’étude québécoise, une distinction est clairement visible dans les résultats où on note un maximum de 57,6 nmol/L d'arsenic total dans le sang des non consommateurs de fruits de mer comparativement à 195 nmol/L chez ceux qui en consomment et dans le sérum le taux de 11,8 nmol/L soit l’équivalent 2,45 µg/L. Cette valeur est presque égale à la valeur médiane des cas.

### Cadmium

Il a été observé des concentrations médianes en cadmium normales dans les deux populations (malnutris et biens nourris) soit respectivement 0,10 et 0,36 µg/L. Nos résultats sont similaires à ceux trouvé par Sherif et al [19] qui lui a trouvé une concentration médiane de 0,37 µg/L chez les enfants normaux et une médiane de 23,105 µg/L chez les enfants avec cancer. A concentration élevée, les troubles comportementaux et neuropsychologiques ont été liés à l′exposition des enfants à ce métal [16].

Benedetti et al; 1994 [20] ont observé une moyenne géométrique de l'ordre de 3 nmol/L soit 0,625µg/L chez des non-fumeurs de la province de Québec. Cette valeur est normale, mais 2 fois plus concentré que nos enfants biens nourris. De même nos concentrations sont faibles par rapport aux résultats Roggi et al. 1995 [21] qui ont trouvé une moyenne géométrique d'environ 0,51 µg/L dans le sang d'une population d'italiens. Curieusement, ils n'ont pas observé de différence significative entre les fumeurs et les non-fumeurs. Silver trouve une valeur en Cadmium dans le sang ≥0,5 µg/L, ce qui est très élevé par rapport à des taux observé dans nos deux populations et il confirme qu'une carence en fer chez les enfants favoriserait l'absorption du cadmium [22].

### Cobalt

Des fortes concentrations de cobalt chez les enfants bien nourris et mal nourris qui ont été respectivement de 2,47 µg/L et 1,49 µg/L. Des taux élevées de cobalts plasmatiques ont été trouvés chez le patient avec l'hépatite alors que leur taux tissulaire hépatique en cobalt était bas. L’étude italienne de Sabbioni et al 1994 [23] a démontré une valeur de référence de 1,4 à 6,8 nmol/L (0,28 à 1,36 µg/L) dans le sérum, ces taux sont faible par rapport aux valeurs observés dans nos deux populations. Tarmaeva, I., 2007 [24] trouve un taux bas en Cobalt, mais son dosage a été réalisé dans les cheveux.

### Chrome

Les concentrations élevées dans nos deux populations (malnutris et biens nourris). L’étude de l'Institut national de santé publique du Québec [18] qui trouve une moyenne géométrique sérique <3nmol/L soit 0,625µg/L, c′est-à-dire inferieur à nos valeurs observées. Sabbioni et al. 1994 [23] dans l’étude italienne montre des taux sériques entre 0,8 et 8 nmol/L (± 2ET) soit 0,166 et 1,66 µg/L ce qui est inférieur aux résultats de notre étude. Pigatto et al, 2010 [25] dans son étude sur les dermatites a pu trouver une part important du chrome dans la survenue de cette maladie mais dans notre étude, nous avons trouvé des cas des dermatoses, mais nous n'avons pas cherché une causalité avec le taux de chrome. L’étude de Remy et al; 2014 [26] démontre que la présence du chrome dans la fumée peut d’être responsable de la survenu des cancers, aussi le chrome peut altérer la réponse immunitaire et serait aussi impliqué dans la survenu des maladies respiratoires. Contrairement à notre étude, nous ne savons pas dire si nos maladies respiratoires ont été liées à un taux élevé en chrome.

### Cuivre

Dans cet étude, nous avons constaté des taux médian bas en cuivre chez les malnutris comme chez les biens nourris avec respectivement de 91,38µg/L et 68,28 µg/L. Ce constat est identique à celui rapportait par Lungambi et Mbensa 1990 [27] qui ont trouvé, chez 90 enfants atteints de kwashiorkor, une hypocuprémie chez la quasi-totalité des enfants avec des taux sériques de l'ordre de 76,77 ± 18,70µg/L. Yones 2015 [28], trouve des taux de 62, 45± 14,81µg/L et 55,68 ±13,94 µg/dl chez les enfants infectés et non infectés respectivement. Arredondo et al, 2014 [29] ont trouvé la concentration moyenne de cuivre sérique chez les enfants malnutris en dessous de la coupure à l′admission à l′hôpital et augmenté après 15 jours (test t, p <0,01). Tarmaeva 2007 [24] trouve un taux bas en cuivre, mais son dosage a été réalisé dans les cheveux. Nash& Mowatt 1993 [30] trouvent des taux bas en zinc et autres métaux chez des enfants présentant une giardiase ou une malnutrition.

### Fer

Dans les deux populations de notre étude il a été observé des taux bas de fer, mais plus bas chez les enfants malnutris. La carence en Fer pourrait protéger contre les infections causées par divers microorganismes, y compris des parasites [31]. Nos résultats sont contraires à l’étude de Shindano 2006 [32] sur la malnutrition sévère œdémateuse de Kapolowe, laquelle était caractérisée par des réserves martiales relativement importantes coexistant avec l'anémie. Par contre, nos résultats sont similaires, à ceux trouvés par Berger; 2006 [33] en Thaïlande qui dans une étude sur des enfants malnutris a fait le constat selon lequel 60% des enfants avaient une carence en fer. L'absorption du fer (Fer) est diminuée par le manganèse [34]. Zanin, 2015 [35] dans une étude effectuée au Brésil, trouve les taux de prévalence de carence en fer étaient de 18,4% (IC à 95% 14.7- 22,6) et 21,8% (IC à 95% de 17,8 à 26,2), et le taux d′incidence de la carence en fer et l′anémie a été de 3,2% et 21,8%. Ces taux sont très faibles par rapport à nos observations.

### Manganèse

Dans cette étude, il a été constaté un taux très bas de manganèse chez les enfants malnutris (0,0002µg/L) par rapport à la valeur normal (2,5µg/L) et par rapport aux enfants biens nourris (2,6µg/L); chez qui, il a été observé une valeur médiane proche de la valeur de référence. D'autres auteurs rapportent des concentrations élevées qui seraient associées à des troubles de comportement et neuropsychologiques [16, 17]. Tarmaeva 2007 [24] trouve un taux bas en manganèse, mais son dosage a été réalisé dans les cheveux. Il y avait une différence significative entre les niveaux de Mn mesurée dans le groupe Marasme (1,582 +/- 0,673µg / L), et le groupe Kwarshiokor (1,811 +/- 0,700 µg / L) et les niveaux obtenus dans les contrôles (3,212 + / - 1 247 µg /L). Lorsqu'ils ont comparé la concentration de Manganèse par sexe ou l′âge dans chaque groupe, ils n′ont pas trouvé de différence significative. Ils ont conclu que les enfants malnutris sévères dans leur étude présentent des concentrations plus faibles de manganèse par rapport aux biens nourris [36].

Weber et al; 1990 [37] ont étudiés les oligoéléments chez les enfants malnutris et ceux en bonne état nutritionnel et il a constaté que les concentrations en manganèse, magnésium et calcium n’étaient pas significativement différents entre les enfants sains et ceux souffrant de malnutrition.

### Magnésium

La valeur observée chez les enfants malnutris est très basse par rapport à la valeur de référence et aussi faible par rapport à la valeur observée chez les enfants biens nourris; chez qui, il a été observé un taux normal par rapport à la valeur de référence soit 18,12µg/L. Ce constat est identique aux résultats que rapporte Kingstone en 1973 [38] qui a trouvé des taux plus bas de magnésium. Au Cameroun, l'hypomagnésémie (<2mg/100 ml) a été observé dans 1/3 des cas de marasme et dans la moitié des cas dans les autres groupes [39]. Amare et al 2012; [40] trouvent que une moyenne sérique de magnésium de 2,42 ± 0,32 (µg / dl) avec une carence en magnésium de 2% chez les enfants d′âge scolaire. Kedzierska et al 2002 [41] dans son étude a trouvé que les concentrations de magnésium dans le sérum et les érythrocytes étaient en dessous de la fourchette normale, mais a été dans la norme dans les cheveux. Les concentrations moyennes de magnésium total dans le sérum, les érythrocytes, et ses cheveux ont été de 0,69 mmol / L (1,7 µg / dL), 1,6 mmol / L ( 3,9 µg / dL) et 0,9 mmol /dL ( 21,5 µg /dL respectivement. 35 enfants parmi eux avaient une hypomagnésémie et des concentrations élevées de plomb et de cadmium ont bénéficié de la supplémentation en magnésium.

### Nickel

Cette étude montre des taux inférieur ou égale à la valeur de référence respectivement pour les malnutris et les biens nourris (1,7315µg/L et 5,655 µg/L). La valeur médiane des malnutris est très faible par rapport à la valeur des biens nourris.

L’étude de l'institut national de santé publique du Québec [18] montre les taux sériques de 17nmol/L soit 3,54µg/L avec un pourcentage de détection de l'ordre de 75%. Sabbioni et al. (1994) [23] observent des valeurs de référence (±2ET de la moyenne) dans l'urine, le sang et le sérum respectivement de 0,20-6,25µg/L, 45-11,6µg/L et 0,8-10µg/L. Pour cette valeur sérique, la limite supérieure observée est deux fois supérieure à la médiane des enfants biens nourris de notre étude et aussi deux fois supérieure à la valeur de référence.

### Plomb

Depuis 1990 la limite réglementaire de déclaration obligatoire du saturnisme infantile a été fixée à 100µg/L. Cependant, les effets du plomb sur la santé sont fonction de l'importance de l'imprégnation. Le taux de 100µg/l correspond au niveau de détection des signes cliniques. Mais il n'y a pas d'effet seuil pour la toxicité du plomb circulant en particulier chez les jeunes enfants et l'OMS [42] donne le seuil de 5 µg/L à partir duquel les manifestations neurologiques et le QI sont affectées. Cependant, certains chercheurs affirment même que toute concentration de plomb dans le corps peut conduire à un dysfonctionnement des processus biochimiques dans le cerveau [43, 44].

Dans cette étude, il n'a pas été constaté une différence significative des niveaux de concentration entre les malnutris et les biens nourris. Soit respectivement 13,07 ±12,06µg/L chez les enfants malnutris et12, 1 ± 11,16µg/L chez les enfants biens nourris. On note une augmentation des niveaux de plomb avec l’âge. Calderón en 2001 [45], trouve au Mexique une valeur proche de la médiane de nos biens nourris soit 9,7 +/- 0,02 (µg / dl) pour le groupe de référence.

Certaines études ont démontré la présence de plomb dans le tabac et l’âge semble jouer un certain rôle [46]. Ceci pourrait être une conséquence de l'utilisation du plomb dans l'essence et la notion de fumeurs passifs chez les enfants il y a plusieurs années. L’étude NHANES démontrent chez 3200 individus, une moyenne géométrique dans le sang de 0,3µg/L. Nos valeurs restent élevés par rapport à l’étude NHANES, mais faibles par rapport à celle de l'enquête de l'institut national de santé publique du Québec [18] qui présente une valeur de 20µg/L. Paolielo et al;1997 [47] ont rapporté dans une étude brésilienne, une médiane de 0,38µmol/L soit 79,16µg/L de plomb dans le sang de 206 sujets non exposé. L'antimoine est récupéré des minerais de cuivre ou se retrouve dans une même carrière que le cuivre. Sabbioni et al. [23] dans leur étude chez les italiens, démontrent des taux urinaires, sanguin et sériques (± 2 ET de la moyenne) respectifs de 0,06-0,13µmol/L (12,5-27,08µg/L), 0,19 - 1,3µmol/L (39,5-270,8µg/L) et 0,0005-0,0024µmol/L (1,04-0,5µg/L). La concentration sérique italienne est très faible par rapport à la plombémie constatée dans la ville de Lubumbashi. Kim et al; 2013 [48] trouve la moyenne géométrique à 1,89 pg / dL et confirme que l′exposition postnatale Pb peut être associée à un risque plus élevé de TDAH clinique. Les principales sources de plomb pour notre population sont le tabac, la poussière contaminée et occasionnellement la vieille peinture au plomb. le plomb est principalement absorbé par voie pulmonaire sous forme de vapeurs, de poussières ou de fumées. La voie digestive est toutefois à considérer soit chez les enfants qui mangent la terre ou de la peinture.

### Antimoine

L'antimoine a été détecté principalement dans le sérum, les taux sériques se sont avérés très élevés dans les analyses de nos deux populations (malnutris et biens nourris). Contrairement à la moyenne géométrique observée par l'institut national de santé publique du Québec [18] qui était <1 nmol/L (0,2µg/L).

Sabbioni et al. [23]. ont répertorié chez 22 sujets italiens des niveaux sériques de 0,1nmol/L à 14 nmol/L (0,020 à 2,9 µg/L). Cornelis et al; 1994 [49] ont documenté une moyenne sérique de 0,11 nmol/L (0,02 µg/L) chez 27 sujets belges. Les valeurs très élevés dans notre étude seraient liées à une contamination; parce que l'antimoine peut être présent dans certains médicaments et dans le tabac. Il est peu présent dans l'environnement général. L'antimoine est récupéré dans les minerais de cuivre ou se retrouve dans une même carrière que le cuivre. L'antimoine pénètre faiblement l'organisme par voies digestive et pulmonaire; il ne s'accumule pas dans l'organisme. Cullen et al 1998 [50] avaient de chiffre très bas de l′antimoine dans le sérum variant entre de 0,09 à 0,25 µg / l (moyenne ± 1.96 SD) chez les nourrissons.

### Sélénium

Dans notre étude, nous avons observé des taux très bas dans les deux populations (malnutris et témoins) soit respectivement 0,0001et 7,04 µg/L. Sabbioni et al 1994 [23] rapportent des valeurs de référence (±2ET de la moyenne) dans l'urine, le sang et le sérum respectives de 0,03-0,4 µmol/L, 0,96-1,8µmol/L et 0,7-1,3 µmol/L (145-270 µg/L). Ce niveau dans le sérum est nettement plus fort ou élevé que le taux détecté dans notre étude. Une étude effectuée sur le statut du sélénium sérique sur 483 écoliers en Espagne au Madrid présente des concentrations de sélénium sérique basse par rapport à nos enfants biens nourris soit une moyenne (DS) 71,1 (14,4) ng L (-1), cependant, était < 60 ng L (-1) dans 13,9% des sujets, et < 45 ng L (-1) à 5,6% et il insiste que bien que l'apport en sélénium est généralement supérieure à celle recommandée, la concentration en sélénium dans le sérum des enfants pourrait être améliorée. Ceci pourrait être réalisé en augmentant la consommation relative de céréales et d'autres aliments riches en sélénium tels que le poisson. Au Brésil, Simone et al; 2014 [51] ont démontré que la malnutrition et le taux de CRP augmenté était associé à un faible taux de sélénium dans plasma. L′interaction entre ces deux variables était significative. Lorsque les valeurs de CRP étaient inférieures ou égales à 40 mg / L, la malnutrition est associée à un bas niveau plasmatique de sélénium (odds ratio (OR) = 3,25, intervalle de confiance à 95% (IC) de 1,39 à 7,63, p= 0,007. Ce constat est similaire à nos résultats, en dehors de taux bas de sélénium, nous avons pu observer cliniquement que la plupart de nos enfants malnutris avaient des infections associées.

### Zinc

Il a été constaté des taux sériques médians très bas en Zinc dans nos deux populations (malnutris et biens nourris) soit respectivement de taux médian 23,76µg/L et 34,67µg/L. Ceci est dû au fait que l'alimentation de ces enfants malnutris ou de la population en générale est souvent carencée en protéines et ou en zinc étant fortement associé aux protéines dans divers aliments de consommation courante.

Le même constat a été observé par Shindano, en 2006 [32] dans une étude sur la malnutrition œdémateuse à Bukavu et à Kapolowe. Yones; 2015 [28], trouve des valeurs variant entre 70,70±15,27µg/L et 81,67±27,61µg/dL dans ces deux populations. En Ethiopie Amare 2012 [40] trouve une moyenne de 86,40 ± 42,40 (µg / dl). Au Cameroun, Ponka R et al 2012 [52] trouvent des valeurs basses chez les écoliers.

## Conclusion

Nous avons constaté que les enfants malnutris et biens nourris présentent tous une malnutrition en micronutriments. Les oligoéléments essentiels sont à des taux très bas dans ces deux groupes d'enfants et qu'ils sont aussi contaminés avec des métaux lourds. Les carences nutritionnelles peuvent aggraver l'impact défavorable de l'exposition environnementale et toxique tels que les métaux lourds qui ne sont pas bénéfique pour le développement. Cette étude nous a permis de déterminer des valeurs pour une gamme d’éléments à Lubumbashi. Les éléments où l'on retrouve les plus importantes modifications, en termes d'ajustement des besoins alimentaires sont: le sélénium, le Zinc, le magnésium, le fer et même le cuivre. Il y a des métaux ou éléments qui sont en excès avec un risque de toxicité: l'antimoine, l'arsenic, le manganèse, le nickel, le plomb et le cadmium; d'où l'intérêt d'une prise en charge spéciale des enfants dans notre milieu vu le risque de pollution par supplémentation en oligo-éléments essentiels.

### Etat des connaissances actuelle sur le sujet

- L'importance des éléments traces dans la croissance et l'immunité chez l'enfant;
- Les effets néfastes des éléments traces polluants chez l'enfant.

### Contribution de notre étude à la connaissance

- Cette étude a permis de déterminer les taux des oligo-éléments dans la population pédiatrique vivant dans un environnement minier;
- les enfants malnutris et biens nourris présentent tous une malnutrition en micronutriments. Les oligoéléments essentiels sont à des taux très bas dans ces deux groupes d'enfants et qu'ils sont aussi contaminés avec des métaux lourds.
